# Evaluation of the FilmArray® system for detection of *Bacillus anthracis, Francisella tularensis* and *Yersinia pestis*

**DOI:** 10.1111/jam.12107

**Published:** 2013-01-31

**Authors:** DR Seiner, HA Colburn, C Baird, RA Bartholomew, T Straub, K Victry, JR Hutchison, N Valentine, CJ Bruckner-Lea

**Affiliations:** Pacific Northwest National Laboratory, Chemical and Biological Signature Science Group, National Security DirectorateRichland, WA, USA

**Keywords:** biodetection, bioterrorism, biothreat panel, FilmArray®, first responders, hand portable, pathogens, PCR, sample preparation

## Abstract

**Aims:**

To evaluate the sensitivity and specificity of the BioFire Diagnostics FilmArray® system in combination with their Biothreat Panel for the detection of *Bacillus anthracis* (*Ba*), *Francisella tularensis* (*Ft*) and *Yersinia pestis* (*Yp*) DNA, and demonstrate the detection of *Ba* spores.

**Methods and Results:**

DNA samples from *Ba*, *Ft* and *Yp* strains and near-neighbours, and live *Ba* spores were analysed using the FilmArray® Biothreat Panel, a multiplexed PCR-based assay for 17 pathogens and toxins. Sensitivity studies with DNA indicate that the limit of detection is 250 genome equivalents (GEs) per sample or lower. Furthermore, the identification of *Ft*, *Yp* or *Bacillus* species was made in 63 of 72 samples tested at 25 GE or less. With samples containing 25 CFU of *Ba* Sterne spores, at least one of the two possible *Ba* markers was identified in all samples tested. We observed no cross-reactivity with near-neighbour DNAs.

**Conclusions:**

Our results indicate that the FilmArray® Biothreat Panel is a sensitive and selective assay for detecting the genetic signatures of *Ba*, *Ft* and *Yp*.

**Significance and Impact of the Study:**

The FilmArray® platform is a complete sample-to-answer system, combining sample preparation, PCR and data analysis. This system is particularly suited for biothreat testing where samples need to be analysed for multiple biothreats by operators with limited training.

## Introduction

The anthrax attacks of 2001 and the uncovering of recent bioterror plots highlight the importance of biodetection systems that can rapidly and accurately identify a wide range of potential biothreats in environmental samples (Schmitt and Shanker 2011; Kman and Bachmann 2012). An ideal system consistently detects the target organism(s) of interest at low levels without significant false-positive or false-negative results, interrogates a single sample for multiple targets (e.g. multiplexed detection), requires limited training and is cost-effective. One of the promising approaches to meet these needs is a detection system that identifies genetic signatures of biothreat pathogens using polymerase chain reaction (PCR). In this study, we evaluated a PCR-based detection system for the analysis of *Bacillus anthracis* (*Ba*)*, Yersinia pestis* (*Yp*) and *Francisella tularensis* (*Ft*).

Conventional PCR-based systems have several distinct advantages for biothreat detection. PCR is sensitive (as compared to protein-based immunoassay methods), and it is also relatively rapid (as compared to direct culture methods) (Ulrich *et al*. 2006). Furthermore, well-designed PCR primers can selectively amplify target organisms *vs* genetically related near-neighbour organisms, reducing the likelihood of false-positive results (Hoffmaster *et al*. 2002).

However, the use of PCR-based systems is not without drawbacks. For example, PCR sample preparation is typically cumbersome, requiring a dedicated laboratory and trained personnel, particularly for environmental samples, where dirt, dust or other debris may prove challenging. Standard PCR systems are also limited by the number of targets that can be addressed in one reaction; addressing too many targets in a single reaction can decrease the sensitivity or specificity of the overall assay (Grondahl *et al*. 1999). Furthermore, the time required for PCR analysis of a sample (sample to answer) is on the order of 1 h and can be several hours depending on the sample preparation method. This is typically longer than lateral flow immunoassays which can take as little as 15 min, but significantly faster than culturing, which requires hours to days.

There has been a significant amount of research focused on the development and integration of automated sample preparation with PCR analysis for the detection of pathogens (Chandler *et al*. 2001; Hindson *et al*. 2005; Lee *et al*. 2006; Lui *et al*. 2009; Zhang *et al*. 2011; Foudeh *et al*. 2012). There are now hand-portable commercially available PCR systems that include assays for pathogen detection, such as the Bio-Seeq PLUS from Smiths Detection, the T-COR 4*™* from Tetracore and RAZOR® EX and FilmArray® from BioFire Diagnostics Inc. (previously Idaho Technology Inc., Salt Lake City, UT, USA). However, the FilmArray® system is currently the only commercial hand-portable PCR-based detection system for pathogen detection that includes integrated sample preparation.

The FilmArray® system utilizes a ‘Lab-in-a-Pouch’ approach for the sample-to-answer detection of 17 biothreat pathogens in a single sample in just over 1 h. The system uses pouches containing all of the lyophilized reagents required for sample preparation, PCR and end*-*point detection. The biological sample, once in the pouch, is subjected to lysis, followed by DNA separation, purification and two-stage nested PCR (Poritz *et al*. 2011). The system can process liquid samples, so any solid samples such as powders collected on a swab must first be mixed with the supplied buffer solution. Briefly, the sample analysis process includes (Poritz *et al*. 2011): (i) 60-s mechanical disruption by vigorous mixing with ceramic bead along with *Schizosaccharomyces pombe* yeast cells that are freeze-dried within the pouch and serve as the internal control (ii) total nucleic acid isolation using silica-magnetic beads (iii) 3 washes and elution of the nucleic acids from the beads (iv) reverse transcription and first stage PCR (multiplexed) (v) sample dilution and splitting into 120 wells for second-stage (single-plex) PCR and (vi) amplicon melt analysis to measure PCR product in each well. The instrument is controlled by a laptop computer, and the integrated software analyses the data from multiple reaction wells (all reactions are run in triplicate) to determine the presence of a pathogen target.

BioFire Diagnostics has recently received FDA approval for use of the FilmArray® platform with the Respiratory Panel pouch that targets a panel of 15 respiratory pathogens (Poritz *et al*. 2011). The Respiratory Panel has been recently evaluated by several groups that found the panel to be both sensitive and specific, and to be significantly more sensitive than the Luminex xTAG Respiratory Viral Panel (Loeffelholz *et al*. 2011; Rand *et al*. 2011; Babady *et al*. 2012; Hayden *et al*. 2012; Pierce *et al*. 2012; Renaud *et al*. 2012). In addition, the FilmArray® Blood Culture Panel has been evaluated for the rapid and accurate identification of pathogens and antimicrobial resistance directly from blood culture (Blaschke *et al*. 2012). The goal of this study was to evaluate the FilmArray® platform and Biothreat Panel for the selective and specific identification of three potential biothreat agents: *B. anthracis*, *Y. pestis* and *F. tularensis*. The FilmArray® platform detects multiple pathogens in a single reaction, requires limited sample manipulation and training, and is a rapid sample-to-answer instrument. Our results from this study indicate that the FilmArray® may be a useful tool for biodetection applications where a sample must be interrogated for a wide range of potential biothreats.

## Materials and methods

### DNA strain panels

For the purposes of this study, we are defining the term ‘inclusivity’ panel to denote isolates that should be detected and ‘exclusivity’ panel as near neighbours that should not be detected. The genomic inclusivity strain panel included three pathogenic strains each of *Ba*, *Ft* and *Yp*. The genomic exclusivity strain panel—nontarget agents that have the potential to cross-react but should not be detected in the assay—included three *Bacillus* neighbour strains, three *Francisella* neighbour strains and four *Yersinia* neighbour strains (Table 1). Although *Francisella novicida* is defined as an exclusivity strain here, it was only recently reclassified as its own species (*F*. *tularensis novicida* to *F. novicida*) (Larsson *et al*. 2009). Inclusivity panel genomic DNA was obtained from the Critical Reagents Program (CRP) via the Biodefense and Emerging Infectious Research Resources Repository (BEI) and stored at −20°C until use. In some cases, organisms in the exclusivity panel (BSL-1/2) were obtained from American Type Culture Collection (ATCC) or BEI Resources, and genomic DNA was isolated (Qiagen DNeasy blood/tissue kit, Valencia, CA, USA) following the growth and culture of the individual organism using standard microbiological practices, as noted in Table 1. All genomic stocks were quantified using the Invitrogen Quant-iT PicoGreen® assay kit (Invitrogen, Grand Island, NY, USA).

**Table 1:** Strain panels for DNA tested of the FilmArray®

	Target Strains	Near-Neighbour Strains
*B. anthracis*	*B. anthracis* Ames*	*B. cereus* E33L†
	*B. anthracis* BA1035*	*B. cereus* G9241†
	*B. anthracis* Canadian bison*	*B. thuringiensis* 97–27†
*F. tularensis*	*F. tularensis* subsp. holarctica 425*	*F. novicida* U112‡
	*F. tularensis* subsp. holarctica LVS*	*F. philomiragia* Jensen ATCC 25016‡
	*F. tularensis* subsp. tularensis SCHU S4*	*F. philomiragia* Jensen ATCC 25017‡
*Y. pestis*	*Y. pestis* Antiqua*	*Y. enterocolitica* WA†
	*Y. pestis* Java9*	*Y. pseudotuberculosis* YPIII plasmid+§
	*Y. pestis* Harbin35*	*Y. pseudotuberculosis* YPIII plasmid−§
		*Y. ruckeri* YERS012*

* DNA purchased from CRP.

† DNA extracted from cultured organism obtained from BEI.

‡ DNA extracted from cultured organism obtained from ATCC.

§ DNA purchased from BEI.

The working concentrations of the stock nucleic acids were assessed for PCR inhibitors by real-time PCR using an Applied Biosystems® (ABI) (Foster City, CA, USA) 7500 Fast Real-Time PCR System with a genus or species-specific TaqMan® primer and probe set (sequences not shown). Concentrations based on PicoGreen analysis and analysis of the DNA using real-time PCR indicated that the genomic stocks and spores were free of contaminants that could inhibit PCR.

### Preparation of *Bacillus anthracis* Sterne spores

*Bacillus anthracis* Sterne spores were prepared as described previously (Colburn *et al*. 2011). Briefly, the Sterne strain from a glycerol freezer stock was grown in Tryptic soy broth without dextrose overnight at 30°C in a shaker incubator. To generate spores, this vegetative starter culture was inoculated into nutrient sporulating medium broth and placed into a 37°C shaker incubator for 4–6 days. The cultures were microscopically checked for sporulation and washed 4–5 times with 10–15 ml sterile Milli-Q water to remove vegetative cell debris. The washed spores were resuspended in sterile Milli-Q (Millipore, Billierca, MA, USA) water to a concentration of ∼10^8^ CFU ml^−1^. Spore preparations were checked by microscopy to verify there was <5% vegetative cell contamination.

### Sensitivity and specificity testing using genomic DNA

The inclusivity strain panel, isolates or strains of the target organisms that the assay should detect, and exclusivity strain panel, nontarget agents that have the potential to cross-react but should not be detected in the assay, are detailed in Table 1. To estimate the number of genome copies [genome equivalents (GE)] in a given sample, the following conversion factors, based on the genome mass, were used: *Ba* – 187 GEs/pg, *Ft* – 521 GEs/pg, and *Yp* – 213 GEs/pg. The number of GEs per DNA sample tested ranged from 25 000 to only 12·5 copies of inclusivity strain DNA and from 250 000 to 2500 copies of exclusivity strain DNA (Table 2). We randomized the testing series relative to the organism DNA and concentrations at which the organism DNAs were tested. Blanks, consisting of Tris–EDTA buffer in which no DNA was added, were performed after every five samples containing DNA. A total of 224 samples including 60 *Bacillus*, 60 *Francisella*, 66 *Yersinia* and 38 blank samples were analysed for the sensitivity and selectivity evaluations.

**Table 2:** The approximate genome equivalents (GEs) of DNA delivered into the FilmArray® instrument pouch for each DNA sample contained in a 50-μl sample volume

Total GEs* DNA in sample	Estimated GEs of DNA delivered into pouch
250 000†	113 000
25 000†^,^‡	11 300
2500†^,^‡	1130
250‡	113
25‡	11
12·5‡	6

* To estimate the number of genome copies in a given sample, the following conversion factors, based on the genome mass, were used: *Ba* – 187 GEs/pg, *Ft* – 521 GEs/pg, and *Yp* – 213 GEs/pg.

† Number of GEs tested for each near-neighbour DNA sample.

‡ Number of GEs tested for each target DNA sample.

Each DNA stock was tested on the FilmArray® system using their Biothreat Panel (BioFire Diagnostics Inc., Salt Lake City, UT, USA). The system analyses only one sample at a time. Table 3 lists the 17 agents that the Biothreat Panel is manufactured to detect; however, this study only evaluated the FilmArray® and Biothreat Panel for detection of *Ba*, *Ft* and *Yp*. To run a pouch, 1000 μl of hydration solution (provided by BioFire Diagnostics with the Biothreat Panel pouch) was drawn up into the larger of the two syringes (also provided with the Biothreat Panel pouch). The blunt-tipped needle of the syringe was then inserted into the water injection port of the pouch, and the evacuated pouch automatically pulled in the necessary amount of hydration solution. The DNA sample (50 μl) was then mixed with 500 μl sample buffer (provided with the Biothreat Panel pouch) and aspirated into the small syringe (provided with the Biothreat Panel pouch). The blunt-tipped needle of the sample solution syringe was then inserted into the sample injection port of the pouch, and the evacuated pouch automatically pulled 250 μl of the sample solution into the pouch. Consequently, slightly less than half of the original sample was delivered into the pouch (estimated GEs delivered into the pouch are reported in Table 2). The pouch was then placed into the instrument for analysis.

The time to prepare a pouch for analysis was approximately 5 min, and the entire process from sample to answer could be completed in just over 1 h. All DNA sample manipulations and addition of a given sample to the buffer and the FilmArray® pouch were performed in a dead-air box, PCR workstation (AirClean, Raleigh, NC, USA). To mitigate the introduction of contamination during pouch loading, laboratory spaces were wiped clean with bleach, DNA *Away* (Molecular Bioproducts, San Diego,CA, USA) and ethanol before and after testing each day.

### FilmArray® testing using *Bacillus anthracis* Sterne spores

*Bacillus anthracis* Sterne spore samples were stored at 4°C until use. On the day of testing, the spores were diluted to 500 colony forming units (CFU) ml^−1^ in nuclease-free water (Ambion, Austin, TX, USA) as determined by viable plate counts on BHI agar plates (BD, Franklin Lakes, NJ, USA). Similar to the genomic DNA testing, spore stocks consisting of 25 CFU spores in 50 μl were mixed with 500 μl of sample buffer, and approximately 250 μl of the 550 μl solution was drawn into the pouch.

### Analysis of results

The FilmArray® instrument detects a positive result for a target based on the observation of a melt curve for that target PCR amplicon rather than the observed fluorescence crossing a threshold value (Ct) during thermal cycling. The FilmArray® software provides automated analysis of the data. If the included internal controls provide acceptable results, a ‘call’ is made for each biothreat target. For the detection of virulent *Ba,* the chromosome target (chrom), pXO1 plasmid and pXO2 plasmid must all be detected. If only one or two of these targets are detected, the built-in algorithm returns a positive call for *Bacillus spp.,* but not *Ba*. Two targets are available for the detection of *Ft;* however, only one target needs to be amplified for a call of *Ft*. Likewise, for *Yp,* only one of the two available targets is required for a call of *Yp*. The software also allows the user to view the signatures that are detected for each sample, so we could assess if the system ‘calls’ were the expected results for each pathogen and near-neighbour DNA sample that we tested.

## Results

### Evaluation of the *Ba*, *Ft* and *Yp* assay sensitivities with purified DNA

The FilmArray® Biothreat Panel was recently developed by BioFire Diagnostics to simultaneously detect the genetic signatures of 17 biological threats (bacterial and viral pathogens and toxins listed in Table 3). The platform uses an enclosed pouch system to extract and purify nucleic acids, amplify target genomic sequences in a nested multiplex PCR and analyse the amplicons in an endpoint-melting curve assay. Our study evaluated the FilmArray® system in combination with the Biothreat Panel pouch for the detection of genomic DNA isolated from *Ba*, *Yp* and *Ft,* as well as live *Ba* Sterne spores.

**Table 3:** The biothreat targets tested using the Biothreat Panel pouch of the FilmArray®

Bacterial targets	Viral targets
*Bacillus anthracis* (3 targets)	Eastern equine encephalitis (EEE) virus
*Brucella spp*.	Ebola virus
*Burkholderia mallei/pseudomallei* (2 targets)	Marburg virus (2 targets)
*Clostridium botulinum*	Orthopox virus
*Coxiella burnetii* (2 targets)	Venezuelan equine encephalitis (VEE) virus (2 targets)
*Francisella tularensis* (2 targets)	Variola major virus
*Staphylococcus aureus*	Western equine encephalitis (WEE) virus
*Rickettsia prowazekii*	
*Yersinia pestis* (2 targets)	Plant: *Ricinus communis*

To determine the sensitivity of the assay, 50-μl samples containing dilutions of purified *Ba*, *Yp* or *Ft* genomic DNA ranging from 25 000 to 12·5 GE were analysed. For each pathogen target, DNA from three different strains—the inclusivity panel, listed in Table 1—was independently tested. For samples at 250 GE and above, six samples were tested (two per inclusivity strain). For samples below 250 GE, 12 samples were tested (four per inclusivity strain).

**Figure 1:**
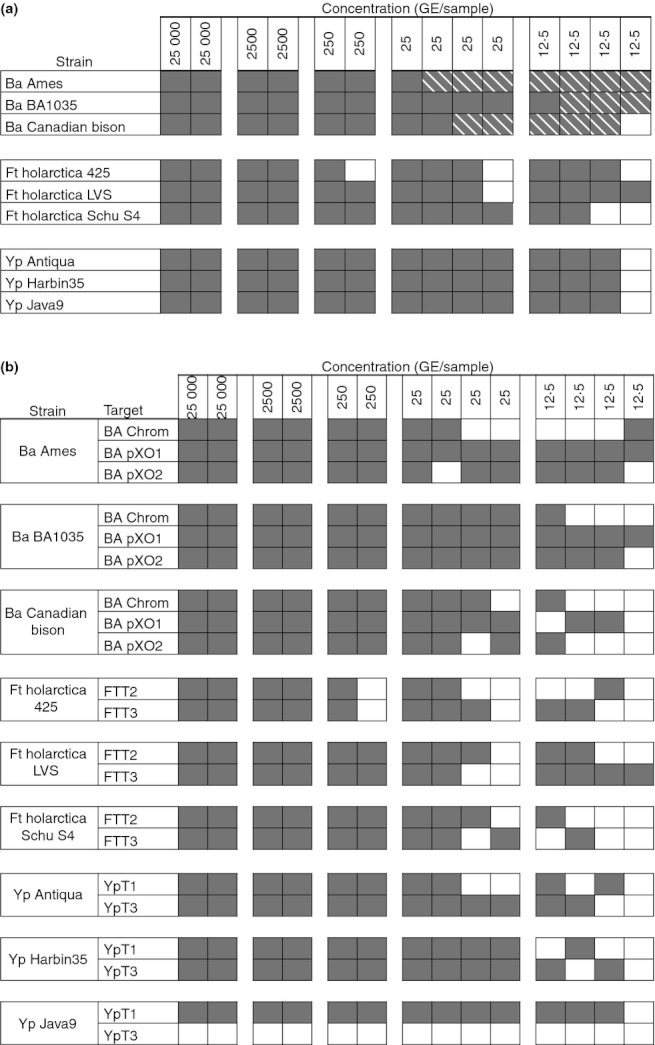
Limit of detection analysis for the *Ba*, *Ft* and *Yp* inclusivity panels. (a) Summary of the instrument calls for each sample. Grey boxes indicate positive and white boxes are negative calls for the target organism; a hatched box indicates an instrument call of ‘*Bacillus* species.’ (b) Test results for individual targets within each test. For the *Ba* test, all three targets must be present for a *Bacillus anthacis* call; if less than three targets are identified, the sample is called as ‘*Bacillus* species.’ For *Francisella tularensis* and *Yersinia pestis*, only one of the targets is required for a call of *Francisella tularensis* or *Yersinia pestis*.

Results from the sensitivity study are summarized in Fig. 1. Figure 1(a) shows the FilmArray^**®**^ system call for each sample, while Fig. 1(b) shows the results of the individual assay targets for each strain. The detection limit for the three target organisms tested was approximately 250 GEs in a 50-μl sample (which was diluted and aspirated into the system as described in the Materials and Methods). Samples containing the target organism at 250–25 000 GEs were called positive in 53 of the 54 samples tested. The one false-negative sample contained *Ft* holarctica 425 at 250 GE. Sensitivity began to drop off for *Ba* with samples below 250 GEs; however, 23 of the 24 samples tested between 25 and 12·5 GEs were called at least *Bacillus* species. For the *Ft* and *Yp* inclusivity samples below 250 GEs, collectively, a positive call was made in 40 of the 48 samples tested.

### Evaluation of the *Ba*, *Ft* and *Yp* assay specificity with purified DNA

To evaluate the Biothreat Panel's specificity, we analysed DNA samples isolated from 9 closely related, but nonpathogenic strains of organisms—the exclusivity panel listed in Table 1—that should not be detected by the assay. The exclusivity samples were tested at concentrations ranging from 2500 to 250 000 GE per 50-μl sample. No unexpected positive results for pathogens (false positives) were observed with any of the 60 exclusivity DNA samples tested (see Figs S1–S4, Supporting Information). One of the exclusivity strains used in this study, *Bacillus cereus* G9241, harbours the pXO1 plasmid and so resulted in positive pX01 signatures, and positive ‘calls’ for *Bacillus spp*. were made but not for *Ba* (as expected). The other two *Bacillus* near-neighbour species in the exclusivity panel do not contain sequence homologous to the chrom, pXO1 or pXO2 signatures and were negative for the *Bacillus spp*. assay as expected. In addition, our results also confirmed that the *Ft* assay is not able to distinguish *F. novicida* from *F. tularensis* (which is an expected result due to the assay design).

Blank samples containing no DNA were also tested. There appeared to be one positive result out of 38 blank samples tested (see Supporting Information). The raw amplification trace and Ct from this blank pouch shows that there was no amplification during the PCR. However, the erratic melt curves were interpreted as positive by the system software.

We did observe some unexpected calls in several samples. Although there were no pathogen false positives in near-neighbour samples, there were four positive calls for *Bacillus spp*. when testing *Yersinia spp*. (either target or near-neighbour). In all of these cases, only one marker for *Ba* was detected. Thus, the multiplexed assay did not produce positive calls for pathogenic *Ba*, which requires all three markers to be positive. These *Yp* assays provided the correct results for *Yp* analysis depending on the species tested (whether a *Yp* target or near-neighbour). In addition, one positive *Ft* sample (*Ft* holarctica 425 at 25 000 GEs) was also positive for *Staphylococcus aureus* (*SA*). Therefore, this sample was shown as positive for two biothreats rather than one biothreat.

### *Bacillus anthracis* Sterne spore testing

To explore the ability of the FilmArray® system to successfully lyse and detect *Bacillus* spores, we conducted limited testing of the instrument with *B. anthracis* Sterne spores. This testing closer mimics a real-world sample collected for biodetection, where the sample is in spore form requiring sample preparation (e.g. lysis) prior to detection. We performed preliminary testing of the Biothreat Panel for spore analysis, with six replicates of 25 CFU spores per 50-μl sample (results reported in Fig. S5). *Bacillus anthracis* Sterne contains the pX01 plasmid, but not the pX02 plasmid (Chen *et al*. 2003). Thus, we expected that the instrument would return a call of *Bacillus spp*. for the detection of the chromosome and pXO1 genetic signatures. In our spore testing, all six replicates were positive for either the chromosome or pXO1 with only 25 spores in the original sample, and three of the 6 samples were positive for both targets. The pX01 assay was positive in five of the six replicates, and the chromosomal assay was positive in four of the six replicates. The pX02 assay was negative for all six replicates, as expected. These results demonstrate the potential field-based utility of the instrument for the analysis of intact spore samples.

## Discussion

Advances in PCR technology over the last few years have led to sensitive and rapid methods for detection; however, there are few fully automated systems for highly multiplexed detection of biothreat agents. Sample-to-answer systems incorporate sample preparation, which can simplify the assay process and reduce the overall analysis time. These systems require less manual sample manipulation, which reduces human error that can occur during sample transfer and pipetting. Simple sample-to-answer systems can also decrease the amount of training required for instrument use. Unlike other sample-to-answer PCR systems that have been developed, the FilmArray® is the first highly multiplexed sample-to-answer PCR biothreat detection system.

Overall, we observed that the FilmArray® consistently detected 250–25 000 GEs of *Ba*, *Ft* or *Yp* genomic DNA in a sample and that detection in 50% or more of the samples occurred at lower concentrations (25 and 12·5 GEs per sample). As we noted in the Materials and Methods section and Table 2, the Biothreat Panel pouch analyses less than half of the available sample (approximately 250 μl of the 550 μl diluted sample is drawn into the pouch). Therefore, it is conceivable that the sensitivity could be improved even further by optimizing the method for introducing the sample into the system.

We observed one of the *Yp* inclusivity strains, *Y. pestis* Java 9, was consistently negative for the *Yp*T3 signature, even in the most concentrated samples. This strain, unlike the other *Yp* inclusivity strains tested, lacks the pMT1 plasmid (Tomaso *et al*. 2003). We therefore infer that the YpT3 signature is likely derived from the pMT1 plasmid. Regardless of the specific assay signatures, as only one *Yp* signature is required for a call of *Yp*, the instrument software still identified these samples appropriately as *Yp* in all cases.

As noted above, the Biothreat Panel is unable to currently distinguish between *F. novicida* and *F. tularensis;* however, this is not surprising, as there has been some considerable disagreement in the literature recently regarding the classification of *F. novicida* as a separate species and not as a subspecies of *F. tularensis* (Larsson *et al*. 2009; Busse *et al*. 2010; Huber *et al*. 2010). Further development of the *Ft* assays used in the Biothreat Panel pouch would be needed to distinguish these two near neighbours using the FilmArray® system.

It is also notable that we observed no pathogen false positives in the exclusivity genomic DNA samples, and we observed only two pathogen false positives in all 224 samples analysed (0·9% error rate). One of these false positives was easily determined to be due to a pouch failure of a blank sample, and the other was a *Francisella tularensis* sample that correctly called *Francisella tularensis* (true positive) but incorrectly called *S. aureus* (false positive) in the same sample. We also observed four samples with a positive *Ba* marker when testing *Yersinia spp*.; however, these did not result in *Ba-*positive calls, because all 3 *Ba* signatures are required for a call of *Ba*. These results demonstrate the value of having multiple signatures to increase the confidence of an analysis. Finally, it is also worth noting that of 226 total runs, only two runs did not report results, leaving us with a data set of 224 genomic samples. These two cases were due to software crashes (data not shown), and the analysis was repeated with a replicate sample.

Finally, although it is impossible to draw significant conclusions about the utility of the system for spore analysis from the limited number of spore sample replicates in our preliminary spore sample study, it is worth noting that the FilmArray® was able to detect spore samples at extremely low levels (25 CFU per sample). The intended use of this instrument is to provide a sample-to-answer result, which requires both efficient lysis and nucleic acid purification. Spore testing provides a more real-world test case for environmental sample biodetection scenarios, as these samples (e.g. white powder samples) are more likely to be in spore and/or vegetative cell form than purified DNA. The positive results for spore analysis demonstrate the utility of the system for a simulated sample-to-answer situation. Future testing with complex surface and powder samples will provide valuable information about the utility of this system for test case samples.

In conclusion, our testing found that the FilmArray® Biothreat Panel provided both sensitive and selective detection of *B. anthracis*, *F. tularensis* and *Y. pestis* genomic DNA. The assays were highly selective, even at very high concentrations of near-neighbour genomic DNA. Our initial evaluation suggests the FilmArray® Biothreat Panel offers a highly multiplexed detection system that meets many of the essential needs in environmental sample biodetection situations, including, but not limited to: (i) minimal hands-on manipulation of the sample (ii) integrated sample processing (iii) multiplexed detection and (iv) easy interpretation of the results. We are continuing our evaluation of the platform with additional live spore samples in the presence of environmental matrices and potential interferents.

## Supporting Information

**Figure S1** The full results of our *Bacillus* genomic DNA testing. Grey boxes indicate an expected and measured positive result, and a circle indicates an expected positive that was not detected. Light grey boxes with a star indicate positive results for the exclusivity strains that were expected and measured.

**Figure S2** The full results of our *Francisella* genomic DNA testing. Grey boxes indicate an expected and measured positive result, grey boxes with an ‘x’ indicate a false positive, and a circle indicates an expected positive that was not detected. Light grey boxes with a star indicate positive results for the exclusivity strains that were expected and measured.

**Figure S3** The full results of our *Yersinia* genomic DNA testing. Grey boxes indicate an expected and measured positive result, grey boxes with an ‘x’ indicate a false positive, and a circle indicates an expected positive that was not detected.

**Figure S4** The full results of our blank testing. Grey boxes with and ‘x’ indicate a false-positive result.

**Figure S5** The results of our live spore testing. Grey boxes indicate a positive call, while x indicates the target was not detected.
