# Supplementary material for: Evaluation of the FilmArray® system for detection of Bacillus anthracis, Francisella tularensis and Yersinia pestis
Source: J Appl Microbiol. 2013 Jan 31;114(4):992–1000. doi: 10.1111/jam.12107 (PMC3617465; doi:10.1111/jam.12107)
Supplement: Supplementary file 1 [file jam0114-0992-SD1.docx]

**SUPPORTING INFORMATION**

Figures detailing results for genomic tests with *Bacillus, Francisella* and *Yersinia*, as well as spore testing results with *Bacillus* are included as Figures S1, S2, S3, S4 and S5 on the following pages.**Figure S1.** **The full results of our *Bacillus* genomic DNA testing.** Grey boxes indicate an expected and measured positive result, ~~grey boxes with an ‘x’ indicate a false positive,~~ and a circle indicates an expected positive that was not detected. Light grey boxes with a star indicate positive results for the exclusivity strains that were expected and measured.

**Figure S2.** **The full results of our *Francisella* genomic DNA testing.** Grey boxes indicate an expected and measured positive result, grey boxes with an ‘x’ indicate a false positive, and a circle indicates an expected positive that was not detected. Light grey boxes with a star indicate positive results for the exclusivity strains that were expected and measured.

**Figure S3. The full results of our *Yersinia* genomic DNA testing.** Grey boxes indicate an expected and measured positive result, grey boxes with an ‘x’ indicate a false positive, and a circle indicates an expected positive that was not detected.

**Figure S4. The full results of our Blank testing.** Grey boxes with and ‘x’ indicate a false positive result.

**Figure S5. The results of our live spore testing.** Grey boxes indicate a positive call, while x indicates the target was not detected.

| *B. anthracis*  Sterne spores | *Ba* Chrom | *Ba* pX01 |
| --- | --- | --- |
| 25 CFU |  |  |
| 25 CFU |  |  |
| 25 CFU |  |  |
| 25 CFU |  |  |
| 25 CFU |  |  |
| 25 CFU |  |  |
